# Characterization of an Unexpected μ_3_ Adsorption of Molecular Oxygen on Ag(100) with Low-Temperature STM

**DOI:** 10.1021/acs.jpcc.4c06572

**Published:** 2024-12-31

**Authors:** Merve Ercelik, Andrés Pinar Solé, Liang Zhang, Piotr Kot, Jinkyung Kim, Jungseok Chae, Lukas E. Spree, Hua Guo, Andreas J. Heinrich, Yujeong Bae, Dmitriy Borodin

**Affiliations:** † Center for Quantum Nanoscience, Institute for Basic Science, Seoul 03760, South Korea; ‡ Ewha Womans University, Seoul 03760, South Korea; § Department of Chemistry and Chemical Biology, Center for Computational Chemistry, University of New Mexico, Albuquerque, New Mexico 87131, United States

## Abstract

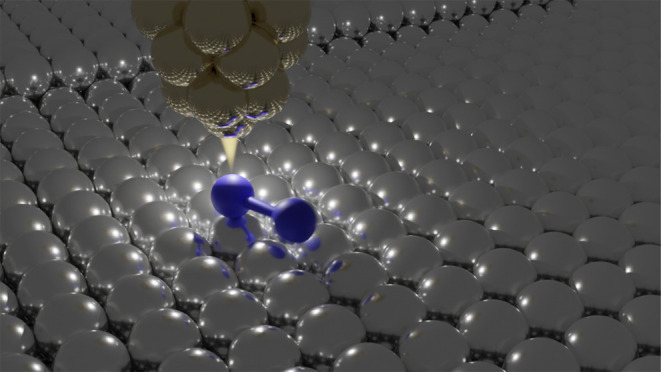

Precise description of the interaction between molecular oxygen and metal surfaces is one of the most challenging topics in quantum chemistry. In this work, we use low-temperature scanning tunneling microscopy (STM) to identify and characterize an adsorption state of molecular oxygen that coordinates to three Ag atoms (μ_3_) on Ag(100). Surprisingly, μ_3_-O_2_ cannot be identified as a stable configuration with generalized gradient approximation (GGA)-level density functional theory (DFT) calculations. Through inelastic electron tunneling spectroscopy (IETS), we identify three vibrational modes of individual μ_3_-O_2_ and assign them to out-of-plane hindered rotation (HR) at 38.0 meV, in-plane HR at 32.4 meV, and in-plane hindered translation (HT) at 22.0 meV. We determine the barrier for rotational isomerization of μ_3_-O_2_ to be 69.3 meV from tunneling electrons-induced rotations. The inability of theory to predict the experiment stems most likely from self-interaction errors inherent to GGA-DFT, which leads to an inaccurate description of localized charges. We speculate that the μ_3_-O_2_ configuration represents a formal molecular oxygen anion and assign the ±11 meV excitation in the IETS to a transition between spin–orbit states of the surface-bound anion.

## 1 Introduction

Understanding the interaction of molecules with metal surfaces is essential to describe chemical reactions on surfaces, which are fundamental for heterogeneous catalysis and other chemical processes at the gas–surface interface. Over the past decades, computational advances have made quantum chemistry the main driving force for the exploration and optimization of catalytic processes.^1−3^ Today, almost all electronic structure calculations for catalytic processes rely on density functional theory (DFT), mostly at the level of generalized gradient approximation (GGA). Despite its overall success, GGA-DFT has some crucial limitations.^4,5^ The interaction of molecular oxygen (O_2_) with metal surfaces takes the leading role in showcasing the limitations of established electronic structure theory in surface chemistry.

Unlike many other molecules involved in important catalytic processes, O_2_ has two unpaired electrons in its ground electronic state (*X*^3^∑_*g*_^–^). This has raised the important question of whether its spin may determine chemical reactivity on metals, as shown for chemical reactions in the gas phase.^6−8^ For example, low dissociation probabilities of O_2_ on Al(111) were initially interpreted by spin selection.^9^ Experiments found that dissociative adsorption becomes more likely as the translational energy of impinging molecules increases,^10^ clearly indicating a barrier for the reaction. However, no barrier was found from adiabatic GGA-level DFT calculations. To resolve the discrepancy between experiment and theory, Behler et al. proposed a spin-restricted model, where the entire molecule–surface system was forced to remain on the triplet state potential energy surface (PES).^9^ In the triplet state, O_2_ dissociation proceeds over a barrier, and the corresponding molecular dynamics simulations yielded reasonable agreement with the experiment.^11^ However, O_2_ also has a high electron affinity (+0.45 eV) which distinguishes it from many other stable molecules in catalysis. A high electron affinity increases the likelihood of charge transfer to the molecule when it is bound to a metal. Unfortunately, GGA-level DFT has large inaccuracies when describing charge transfer, as the semilocal exchange-correlation functionals prefer charge delocalization.^5^ This situation has raised doubt about the proposed spin selection rules. In fact, Carter and co-workers^12^ showed that an adiabatic barrier for O_2_ dissociation on Al(111) emerges naturally as a consequence of charge transfer. Using embedded correlated wave function (ECW) theory, they could avoid the charge delocalization issues of GGA-DFT. Subsequent molecular dynamics simulations from Jiang and co-workers on an ECW PES quantitatively reproduced the dissociative adsorption probabilities found in the experiment.^13^ At present, the majority of the scientific community seems to have converged on the charge transfer interpretation for this system.^4,13^

The interaction of O_2_ with silver is another interesting combination due to its role in the industrial production of ethylene oxide.^14^ Compared to other surface facets of silver,^14−18^ the interaction of O_2_ with Ag(100) has received little attention, leaving several questions unresolved. For example, upon thermal dissociation of O_2_, the resulting oxygen-atom pairs are found in a broad distribution of distances up to 10 nm via scanning tunneling microscopy (STM).^19^ This observation has been interpreted by the “hot” atom mechanism,^20^ where nascent O atoms receive kinetic energy in the lateral directions during the bond dissociation process. However, molecular dynamics simulations have been unable to reproduce the experimentally observed large lateral distances^21^ and dissociation of O_2_ by tunneling electrons in an STM does not seem to result in large O atom separations either.^22^ Furthermore, several studies of thermal dissociative adsorption suggested that oxygen may proceed through various molecular adsorption states—the so-called “precursor” states—prior to dissociation.^14,15,17^ The involvement of such molecular states may explain the conflicting results between experiments and theory. Identifying and understanding the nature of molecular “precursor” states may therefore be a prerequisite to understanding chemical reactivity in this system. In this context, several adsorption configurations have been proposed for O_2_ on Ag(100)^23^ and high-resolution electron energy loss spectroscopy (HREELS) has identified the presence of two possible O_2_ binding sites.^24^ It is an established procedure to assign surface binding sites through a comparison of vibrational frequencies of adsorbates to frequencies found in multicore metal complexes.^25^ Nevertheless, known examples of such comparisons led to incorrect binding site assignments.^26−28^ Thus, the proposed binding configurations for O_2_ on Ag(100) remain ambiguous, making a real space image highly desirable. Despite previous STM studies on O_2_ assemblies on Ag(100), the binding sites of individual oxygen molecules remain largely unknown.

The examples above demonstrate the challenges that theory faces in describing even the simplest chemical reactions on metal surfaces. It also highlights how crucial experiments can be to understand the fundamental principles of surface chemistry. It is important to realize that simulating reaction probabilities and reactive events requires several subsequent steps, involving electronic structure calculations of the PES and molecular dynamics simulations. Each step may involve a different flavor of approximation, making it difficult to identify the source of experiment-theory disagreement. In the worst case, experiment and theory only agree due to error compensation between several subsequent steps. To test an electronic structure method more specifically, benchmark experimental data such as stable binding sites of molecules, site-specific vibrational frequencies, binding energies, and diffusion barriers should be measured.^29^ This idea serves as the leitmotiv for the present work.

In this work, using low-temperature STM, we identify and characterize a previously unknown adsorption state of adsorbed O_2_ on Ag(100). Molecular oxygen is found to localize close to the center of the 4-fold hollow site with one O atom coordinated to a bridge binding site and the other to an opposite top-binding site, inducing an asymmetric μ_3_-configuration. GGA-level DFT calculations only find symmetric configurations at the 4-fold hollow (μ_4_-O_2_) and the bridge (μ_2_-O_2_) sites, failing to reproduce the experimentally observed μ_3_-O_2_. Using inelastic electron tunneling spectroscopy^30^ (IETS), we observed three vibrational excitations, which we assigned to out-of-plane hindered rotation (HR, 38.0 meV), in-plane HR (32.4 meV), and in-plane hindered translation (HT, 22.0 meV). We find that tunneling electrons promote a configurational change of the μ_3_-O_2_, with a threshold voltage that coincides with the excitation of the in-plane HR mode. This configurational change resembles a “rotation” of the molecule where the O atom at the bridge site jumps to a neighboring and equivalent bridge site while the other O atom at the top site remains in place. We characterized the bias voltage and tunneling current dependence of this rotation and modeled the results as a combination of a two- and a one-electron process. We confirm these interpretations by modeling the nuclear eigenstates of the in-plane HR of μ_3_-O_2_ in a simple one-dimensional (1D) double-well potential. We speculate that the observed μ_3_-O_2_ configuration is likely a formal molecular oxygen anion (O_2_^–^). We discuss this possibility in terms of energetic stabilization of the anion. We speculate that the broad feature found in the IETS at ±11 meV, which could not be assigned to a vibration, is related to the transition between spin–orbit states ^2^Π_3/2_ and ^2^Π_1/2_ found at ∼20 meV for O_2_^–^ in the gas phase.^31^ We believe that our experimental identification and characterization of the μ_3_-O_2_ configuration on Ag(100) will contribute to the development of more reliable electronic structure methods for electrochemistry and heterogeneous catalysis.

## 2 Methods

The experiments have been performed in a home-built low-temperature STM system.^32^ The single-crystal Ag(100) surface was cleaned in a room-temperature ultrahigh vacuum (UHV) chamber by cycles of Ar^+^ sputtering (2 kV, 10^–5^ mbar, 7.5 μA) and annealing at ∼730 K for 20 min, each. The molecules were deposited from a leak valve onto Ag(100) held at ∼5 K. The leak valve was positioned in line-of-sight with the low-temperature STM stage (∼2 m distance), where the sample surface was fixed at an angle of ∼83° between the deposition axis and surface normal. In this configuration, O_2_ was deposited for 2 min at a steady-state pressure of 10^–6^ mbar in the room-temperature chamber. The average normal energy^33^ of incident O_2_ molecules is ∼1 meV. The resulting coverage of well-separated, individual O_2_ molecules on the silver sample was on the order of 0.1% ML.

All experiments were conducted at 1.5 K. We used a PtIr tip which was coated with silver by poking into the substrate. The inelastic electron tunneling spectra (IETS) were recorded by a lock-in measurement of the first harmonic at an amplitude of 0.5 mV and a frequency of 638 Hz. The modulation frequency was chosen based on a “silent” region of the noise spectrum of the tunneling current with open feedback loop (10 mV, 1 nA). The *z*-set point of all reported IETS spectra is 50 mV and 1 nA. Telegraph noise experiments were conducted in closed-loop conditions, except for cases where the rotation rate exceeded ∼5 rotations per second. Rotation rates obtained from Δ*z* or Δ*I* changes were benchmarked against each other for slow rotation rates and yield indistinguishable results. The Ag(100) lattice orientation was determined from the lattice of an epitaxially grown MgO thin film.^34^ The positions of Ag atoms underneath the O_2_ molecules were calibrated by neighboring Fe atoms, assumed to bind to the 4-fold hollow site of Ag(100), as is the case for Cu(100).^35^ The uncertainties of the exact Ag(100) lattice position—determined from variations in different images—are within 0.2 Å. In this work, we only make conclusions about individual O_2_ molecules that have been found after a cold deposition of O_2_ gas, several nm away from other species on the surface. Species found within 2 nm distance to other adsorbates on the silver surface have not been considered. We excluded the possibility that the observed configuration of oxygen (μ_3_-O_2_) is specific to defects (or comparable) by on-surface manipulation of the molecules across (100) terraces with the tip.

The spin-polarized DFT calculations were carried out using the Vienna Ab initio Simulation Package (VASP).^36,37^ The ionic core–electron interactions were described by the projector-augmented wave method^38^ and the Kohn–Sham valence electronic wave function was expanded in a plane-wave basis set with a kinetic energy cutoff at 400 eV. The Ag(100) surface was modeled by a four-layer slab with a (3 × 3) supercell with the top two layers relaxed. The slabs were separated by a 15 Å vacuum space in the vertical direction. The Brillouin zone was sampled using a 4 × 4 × 1 Γ-centered *k*-points grid mesh. The exchange–correlation effects were described within GGA using the RPBE^39^ and Perdew–Burke–Ernzerhof (PBE)^40^ exchange-correlation functionals. The geometries were optimized using a conjugate-gradient method until the forces acting on each atom were less than 0.2 meV/pm. The climbing image nudged-elastic band (CI-NEB) method was utilized to search the saddle points along the minimal energy pathway.^41^ The harmonic frequencies were obtained from small spatial displacements of the atoms within the adsorbate.

## 3 Results

**Figure 1:**
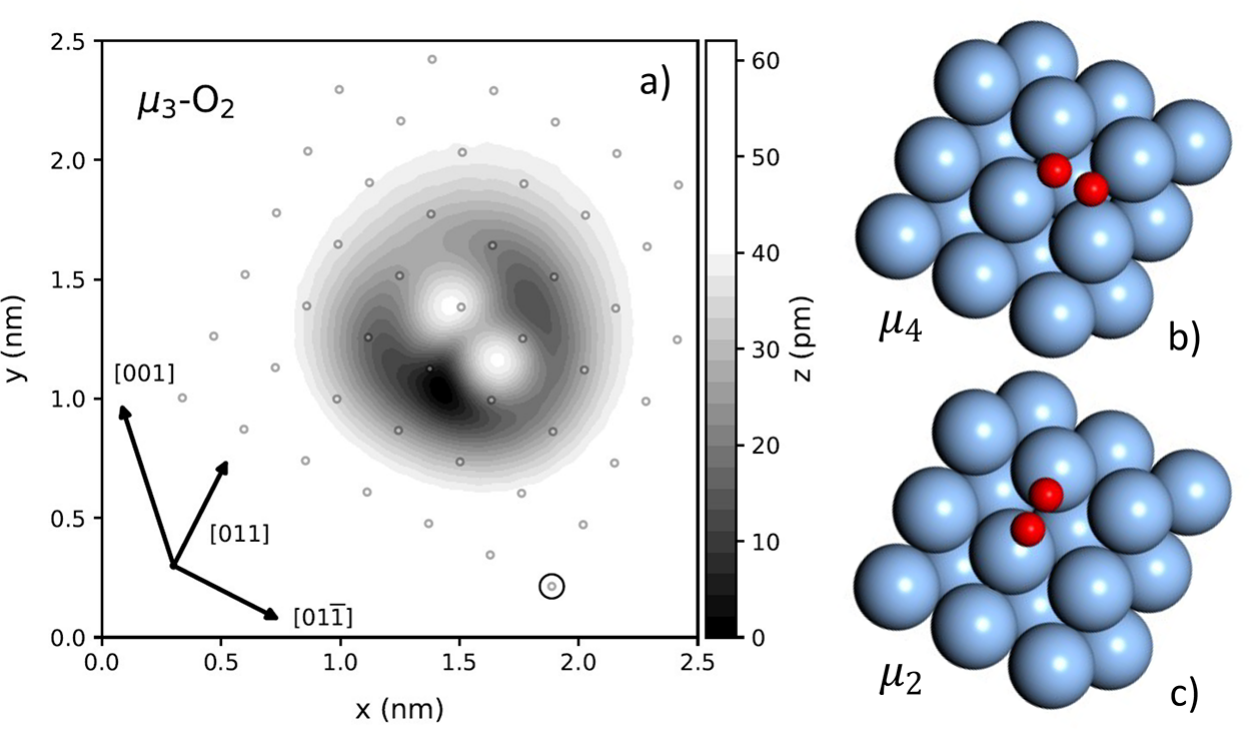
(a) Constant-current STM topography of a single oxygen molecule on Ag(100) acquired at −10 mV and 500 pA. The small gray circles indicate the position of silver atoms, and the large black circle indicates their positional uncertainty with respect to the molecule. The image shows one of the eight equivalent orientations of the oxygen molecule on the lattice (see text). The molecule coordinates to three Ag atoms and we use the bridging ligand nomenclature—μ_3_-O_2_—to distinguish it from configurations in (b) the 4-fold hollow site—μ_4_-O_2_—and (c) the bridge site—μ_2_-O_2_—obtained from DFT.

The STM image in Figure 1a shows a constant-current topography of a single O_2_ molecule found on the Ag(100) surface. The topographies obtained at a conductance of several tens of nS show two lobes of different sizes and two asymmetric depletions. Topographies acquired at a decreasing conductance show less pronounced lobes and converge to slightly asymmetric depletions around 0.5 nS and below, see Figure S1 in the Supporting Information (SI). As previously found for O_2_ on Pt(111), the bright lobes are associated with molecular orbitals, located close to the positions of individual O atoms,^42−44^ allowing us to determine the coordination of the O_2_ on the lattice. We find that one of the O atoms is bound to a bridge site, and the second O atom is bound to an on-top site. We dub this configuration as μ_3_-O_2_ where μ_3_ indicates the number of metal atoms directly binding to the adsorbed molecule. Similar to Figure 1a, we find eight naturally occurring orientations of μ_3_-O_2_ on Ag(100)—compare Figure S2 in the SI. Interestingly, two groups of four orientations exist. Within one group, the configurations can be converted into each other by an integer number of 90° rotations. However, orientations between the groups can only be converted into each other by an additional mirroring operation along [001] or [010] directions, with subsequent rotations, see Figure S3 in the SI. We will show later that conversion between different orientations can be controlled with tunneling electrons as well.

We have performed DFT calculations of O_2_ minimum energy structures on Ag(100). We find two symmetric local minimum energy structures, one at the 4-fold hollow site and the other at the bridge binding site as shown in Figure 1b,c. An antisymmetric configuration, similar to μ_3_-O_2_ observed in the experiment, could not be identified.

**Figure 2:**
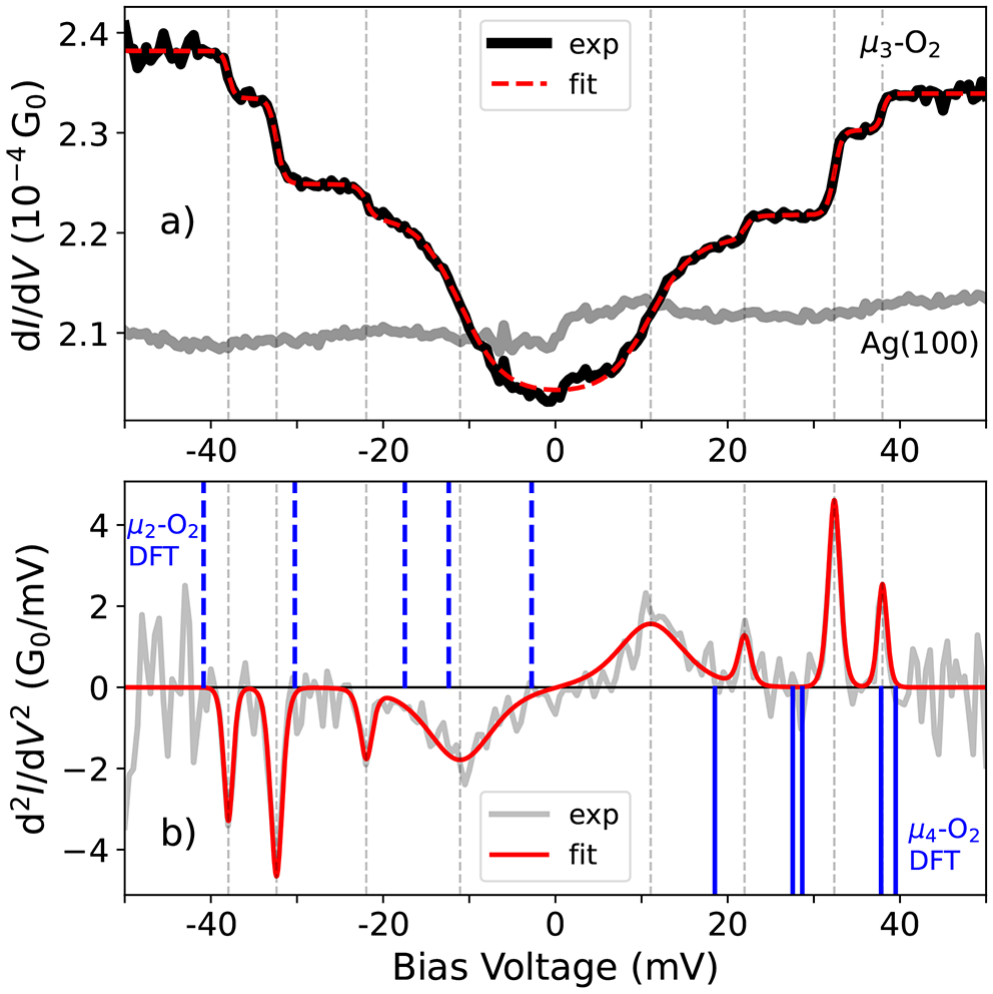
Inelastic electron tunneling spectrum (IETS) for a single μ_3_-O_2_ on Ag(100). (a) Differential conductance (d*I*/d*V*) spectrum obtained from the center of the large lobe (see Figure 3b,c) of μ_3_-O_2_ (black line) and above Ag(100) (gray line). The *z*-set point for both measurements is 50 mV and 1 nA. The step centers at 11.1 ± 0.1, 22.0 ± 0.2, 32.38 ± 0.07, and 38.0 ± 0.1 meV are obtained from the fit (red dashed line) of eq 1 to the black line, as indicated by the gray vertical dashed lines. (b) IETS spectra represented as d^2^*I*/d*V*^2^. The gray and red lines are the numerical derivatives of the black and red dashed lines from (a). The blue solid lines are DFT-derived harmonic frequencies for μ_4_-O_2_ and dashed blue lines for the μ_2_-O_2_ configuration, both using the PBE exchange-correlation functional.

The black line in Figure 2a shows the differential conductance (d*I*/d*V*) spectrum (i.e., IETS) obtained for a single μ_3_-O_2_ on Ag(100). The gray spectrum in Figure 2a shows the d*I*/d*V* spectrum obtained directly on the Ag(100) surface, ∼1 nm away from μ_3_-O_2_. Comparison between gray and black lines shows that the step found around 0 mV in the IETS of the molecule is a feature of the metallic junction, not specific to the molecule. The d*I*/d*V* spectrum of the molecule shows three sharp steps between 20 and 40 mV that are symmetric with respect to bias polarity. Additionally, we observe a much broader, symmetric step around ±11 mV that is distinctly different from the other three excitations.

To determine the observed excitations, we fit a simple model function capable of reproducing the stepped features of the d*I*/d*V* spectrum using Fermi functions
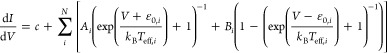
1Here, *c* describes the elastic differential conductance, and *A*_*i*_ and *B*_*i*_ describe the inelastic differential conductance associated with the individual excitation at negative and positive bias, respectively. Using *A*_*i*_ and *B*_*i*_ as independent parameters allows us to account for asymmetries in the IETS. The parameter ε_0,*i*_ describes the bias voltage at the center of the step, and *T*_eff,*i*_ describes the broadening of the excitation, not necessarily the temperature. The fit of the d*I*/d*V* spectrum is shown as the red dashed line in Figure 2a and all optimized parameters for the fit can be found in SI Table S1. The fitted excitations ε_0,*i*_ are at 11.1 ± 0.1, 22.0 ± 0.2, 32.38 ± 0.07 and 38.0 ± 0.1 meV and are indicated in Figure 2 as vertical dashed gray lines. In Figure 2b, we convert the differential conductance spectrum to a d^2^*I*/d*V*^2^ by numerical differentiation of the black line from Figure 2a—gray line—and derivative of the fit function from eq 1—red solid line. Additionally, we add the harmonic frequencies for the μ_4_-O_2_ and μ_2_-O_2_ configurations for comparison in Figure 2b. Although the predicted geometries differ from the μ_3_-O_2_ configuration observed in the experiment, the resulting frequencies are in the right energy range. In the discussion (Section 4.2), we will use the calculated vibrational frequencies to assign the observed excitations.

**Figure 3:**
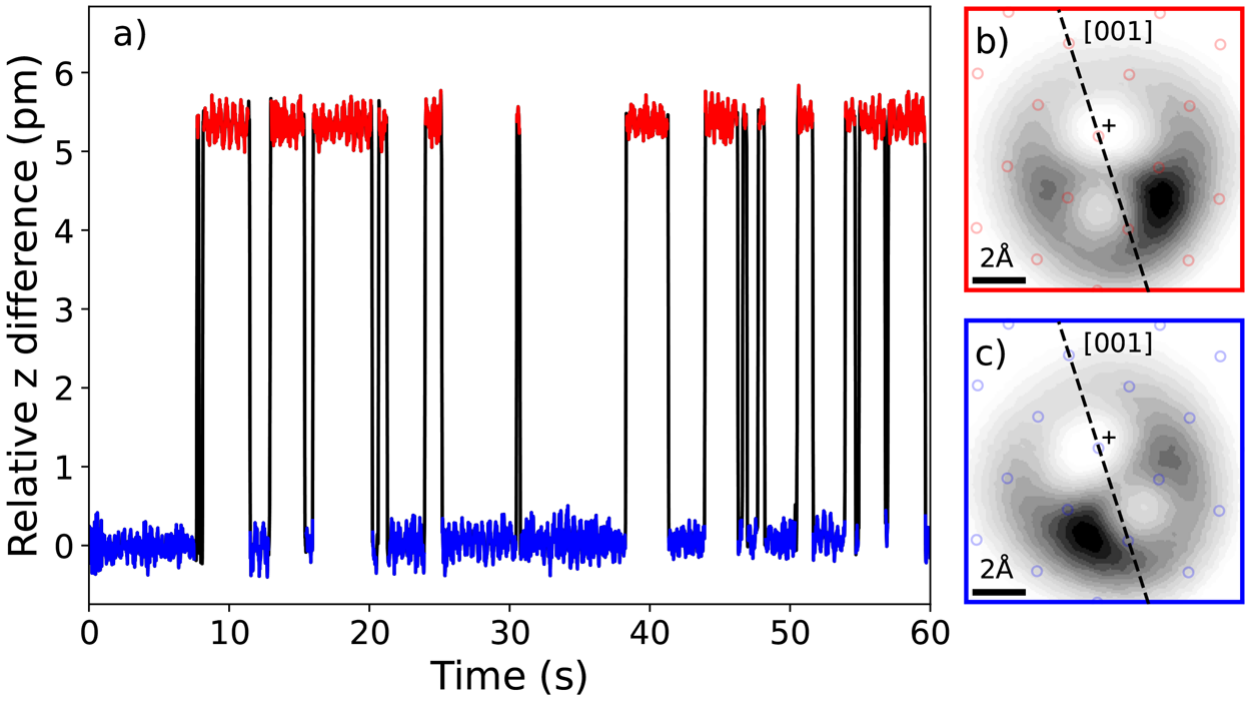
Telegraph noise observed from the electron-induced rotation of a single oxygen molecule on Ag(100). (a) Time-dependent measurement of the *z* height, showing switching between two rotational states. The measurement was conducted in a closed feedback loop at 50 mV and 1 nA. (b, c) Constant-current topographies (−10 mV, 100 pA) of the two rotational isomers of μ_3_-O_2_, which correlate to the two states observed in (a). The black plus in (b) and (c) indicates the STM tip position during the measurement. The circles in (b) and (c) indicate the silver atoms of the surface and the dashed line is the [001] direction, which coincides with the mirror image plane between the two μ_3_-O_2_ conformers.

We observed that at bias voltages of ∼32 mV and higher, the oxygen molecule becomes bistable. Figure 3 presents the telegraph noise observed at a single oxygen molecule adsorbed on the Ag(100) surface, emerging from the electron-induced rotation of the molecule. In Figure 3a, the time-dependent *z* height switching reveals isomerization of μ_3_-O_2_ between two rotational conformers that are shown in panels (b) and (c) with the appropriate color code. The STM topography of rotational conformers was obtained by setting the bias voltage and current such that one switching event occurs every few seconds. At these conditions, the tip is withdrawn by 0.5 nm while the molecule is in one of the discrete states of the telegraph noise trace, effectively freezing the associated configuration. Subsequently, the bias voltage and current were reduced to −10 mV and 100 pA to capture the STM topography. From such data (see Figure 3), we learn that the rotational process involves the jump of the O atom that is coordinated to the bridge binding site to a neighboring bridge site, while the single-coordinated O atom remains in contact with the same Ag-atom and merely has a small shift—compare Figure 3b,c. Both configurations are mirror images of each other, where the mirror image plane is along the [001] axis.

**Figure 4:**
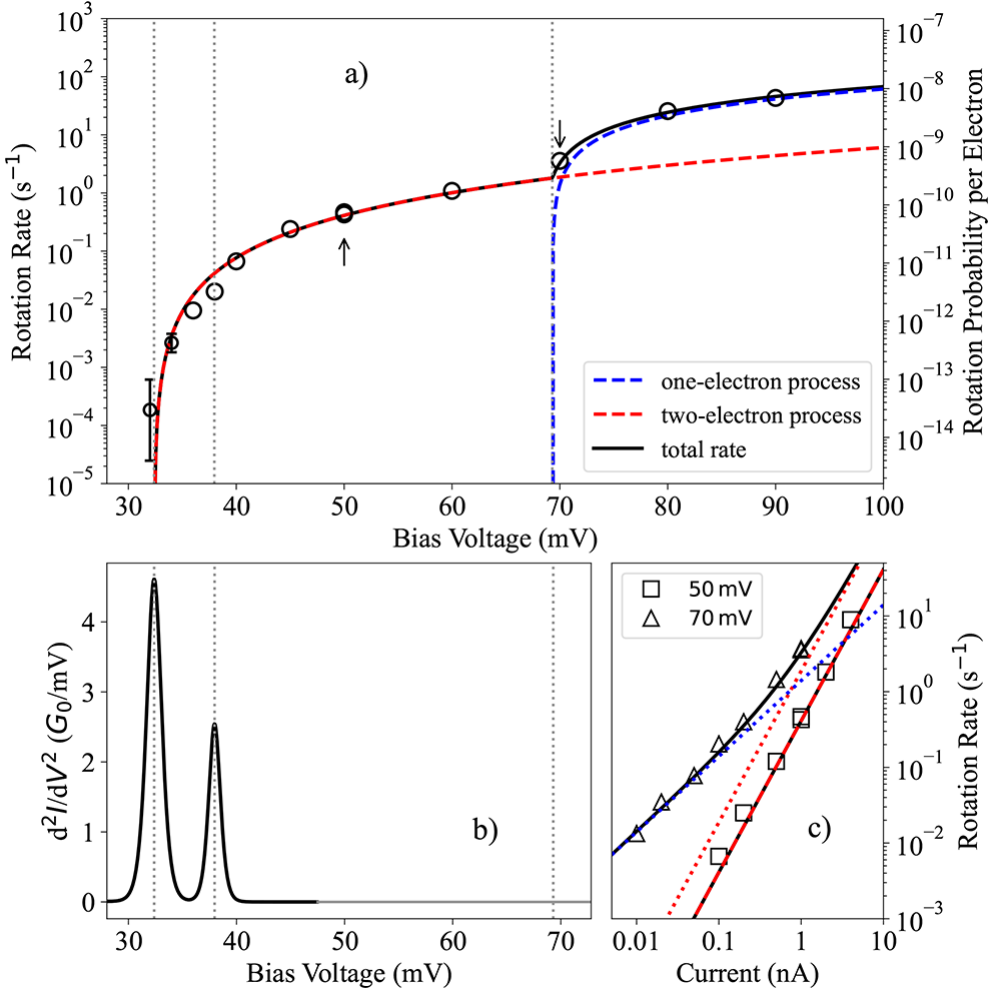
Dependence of rotational rates of a single oxygen molecule at Ag(100) on bias voltage and tunneling current. (a) Rotation rate as a function of bias voltage between 30 and 100 mV at 1 nA tunneling current. The data points at the lowest bias voltages have only few switches and therefore have large error bars, which are estimated from Poisson statistics of small numbers. For all other data points, the error bars are smaller than the size of the marker. Below ∼32.2 mV, not a single rotation is observed for several hours. The arrows indicate the voltages at which the current dependence is measured (c). (b) Vibrational transitions above 30 mV. The peak at 32.4 mV coincides with the threshold enabling the rotational process. (c) Dependence of the rotation rate on tunneling current at bias voltages of 50 and 70 mV. The black lines in (a, c) are based on a rotation rate model including a two-electron (red dashed and dotted lines) and a one-electron (blue dashed and dotted lines) process from eq 2. The model uses a uniform set of parameters between (a, c)—see SI Table S2. Alternative interpretations, involving higher-order processes and additional channels, are excluded but discussed in the SI Section S1.

We measured such switching behavior, commonly referred to as action spectroscopy,^45^ at several bias voltages and tunneling currents, see also Figure S4 in the SI. From the telegraph noise data, we obtained the rotational rate of the molecule by counting the total number of switches per time. The resulting rotation rates as a function of bias voltage and tunneling current are shown in Figure 4. In the voltage range between 30 and 100 mV, we find a monotonic increase of the rotation rate with bias voltage. Below 32.2 mV, we did not observe a single rotation occurring over several hours. We notice that the threshold for electron-induced rotation coincides with the highest peak in the d^2^*I*/d*V*^2^ spectrum—compare Figure 4b—indicating this to be the vibrational excitation for in-plane rotation that enables the isomerization process. We also characterized the dependence of the rotation rate on the tunneling current—see Figure 4c. We find a steeper increase in the rotation rate vs tunneling current at 50 mV, compared to 70 mV, which makes us conclude that the average number of electrons needed to induce a rotation of the molecule is larger at 50 mV than at 70 mV.

Rotation rate measurements at 60 mV and higher show that electron-induced rotation between the states in Figure 3b,c is no longer exclusive and that rotations, where the O atom jumps from one top site to another also become possible—see Figure S5 in the SI. However, due to the low yield of this process, we have not studied it in detail. The increasing rotation rate and the additional rotation process are the main reasons why d*I*/d*V* spectra were challenging to measure beyond ±50 mV.

We interpret the threshold behavior from Figure 4 as tunneling electron-induced isomerization by exciting vibration of the molecule. To better characterize the processes of rotation and determine the number of electrons involved, we model the bias voltage *V* and tunneling current *I* dependence of the rotation rate RR by the equation:
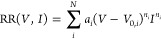
2where *N* is the total number of processes, *i* is the index of the particular process, *a*_*i*_ is a rate coefficient, *V*_0,*i*_ is the threshold voltage, and *n*_*i*_ indicates the number of electrons involved in the particular process *i*. This formula was previously shown to provide a good description of single-molecule reactions and other processes induced by electrons in the limit of low temperatures and sharp density of states.^45^ Our analysis revealed that two processes dominate the rotation rate within the experimental range. The parameters of eq 2 (see Table S2 in the SI) were optimized such that they reproduce the dependence on *V* and *I* simultaneously. From the fitting, we find that the two-electron process is the dominant mechanism for electron-induced rotation up to a threshold voltage of 69.3 mV—see Figure 4a,c. Beyond this threshold, the two-electron process becomes less significant and the total rate is dominated by the one-electron process. Close to the one-electron process threshold at 70 mV, we observe an intricate behavior where the two-electron and one-electron processes compete with each other, depending on the tunneling current—see Figure 4c—a behavior that is perfectly reproduced by our rotation rate model.

## 4 Discussion

### 4.1 Understanding the Thresholds in Rotational Action Spectra

The threshold behavior of the electron-induced rotation near the 32.4 mV peak indicates that the normal mode of vibration is correlated to the isomerization coordinate. Thus, we assign this vibration as the in-plane hindered rotation (HR). We will show an alternative method in Section 4.2 that confirms this assignment. Tunneling electrons can induce a vibrational energy “ladder climbing” that drives the system over the barrier to change its configuration.^16,45−47^ Our analysis shows that a two-electron process dominates the rotation below 69.3 mV, after which a one-electron process takes over. We interpret 69.3 mV as the quantum barrier for rotational isomerization. Other possible interpretations are excluded in this case but carefully discussed in the SI in Section S1.

**Figure 5:**
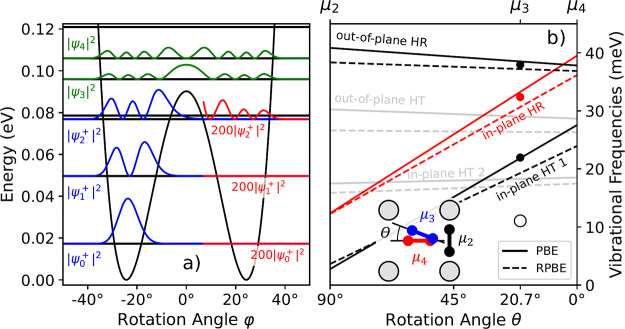
(a) One-dimensional potential (see SI Table S4) describing the electron-induced rotation of μ_3_-O_2_ with the corresponding vibrational energy eigenstates (black lines) and their associated probability densities (blue lines). The eigenstates indicated by a “+” are localized, i.e., specific to an individual configuration, all other states are delocalized over two mirror image conformers (green lines, see also Figure 3b,c). The first two vibrational eigenstates have a weak tunneling splitting and can be approximated as 2-fold degenerate. The delocalization of “+” eigenstates is shown as red lines, which its probability density multiplied by 200 times around the opposite minimum. (b) Walsh diagram illustrates the correlation of DFT-derived vibrational frequencies at different configurations of the oxygen molecule—μ_2_ on the left and μ_4_ on the right. With this correlation, vibrational frequencies observed in IETS of μ_3_-O_2_ (red, black, and open circles) at Ag(100) can be assigned. The drawing in the lower left shows the experimentally observed and DFT-predicted configurations of the molecule. The definition of angle θ is shown in (b) and angle φ is defined in SI Section 2.

To understand whether the magnitude of the observed rotational barrier is reasonable, we make a simple model for a one-dimensional double-well rotational potential. We obtain the energy eigenstates by solving the nuclear Schrödinger equation under the assumption that O_2_ is a one-dimensional rotor—see SI Section S2 for details. We make simplified estimates of the geometric parameters, such as the angle difference between configurations, based on the observed STM topographies—see SI Figure S8—and optimize the classical barrier height to reproduce the vibrational excitation peak observed at 32.4 mV. The rotational potential and the corresponding vibrational levels are shown in Figure 5a. We find only few localized vibrational states that are specific to a single rotational configuration, confirming the low number of electrons required to induce rotation.

We find two vibrational levels close to the classical barrier height—90.2 meV—that can explain the one-electron process threshold, i.e., the rotational barrier, around ∼70 mV as found from the experiment. One state is at 78.7 meV, with respect to the ground vibrational level, and is delocalized between the two minima—|ψ_3_|^2^ in Figure 5a. Excitation into this state, followed by de-excitation into localized eigenstates of the opposite minimum, would likely be a mechanism to induce rotation. Two levels are found below the classical barrier and represent a tunneling doublet with energies between 59.7 and 61.3 meV—|ψ_2_^+^|^2^ and |ψ_2_^–^|^2^ in Figure 5a. The level localized in the initial configuration would have ∼1% probability density on the opposite site of the barrier. Although the probability is low, in principle, if the molecule is excited into this state, there is a finite chance that another electron de-excites the system while on the opposite site of the barrier, leading to tunneling-assisted isomerization. Certainly, the simplicity of our model potential, along with the neglect of possible resonance with other rotational configurations, lifetime broadening or vibrational mode mixing (i.e., Fermi resonances), is the reason that quantitative agreement better than ∼10 mV with the experimental rotational barrier is not reached. Despite these shortcomings, the semiquantitative agreement supports our assignment of the vibrational mode, the rotational barrier, and the mechanism of the electron-stimulated isomerization of μ_3_-O_2_.

For comparison, we have also calculated the barrier for the rotation of the μ_4_-O_2_ configuration with GGA-DFT. We find that the barrier is 0.38 eV, clearly much higher than the barrier for rotation of μ_3_-O_2_ found from experiment. Interestingly, the rotation of μ_4_-O_2_ does not proceed symmetrically around the center of the 4-fold hollow site. Analysis of the minimum energy pathway for μ_4_-O_2_ rotation reveals that the geometries of the O_2_ molecule along the rotation pathway strongly resemble the μ_3_-O_2_ configuration observed in the experiment—see SI Figure S9. However, these configurations are not found to be local minima and are ∼0.2 eV higher in energy than the μ_4_-O_2_ configuration.

### 4.2 Assignment of Vibrational Excitations and Comparison to Previous Work

A free diatomic molecule has six degrees of freedom: three translational, two rotational, and one stretching vibration of its bond. The degrees of freedom for translation and rotation become hindered vibrations due to the interaction with the surface. The stretching frequency of the oxygen molecule is expected to be higher than 50 meV and could not be observed in our experiment due to the high rotational activity of the molecule during IETS experiments above ∼40 mV. GGA-DFT calculations show that HR and hindered translational (HT) modes are within 50 meV for the μ_4_-O_2_ and μ_2_-O_2_ configurations. Thus, we may expect up to five vibrations in a similar range. We have identified the in-plane HR mode by comparison to electron-induced rotational rates. We observe two additional excitations in the IETS at ∼22 and ∼38 meV that have similar widths but slightly lower intensity—see Figure 2. These are likely additional vibrations. In addition, we observe a broader peak at ∼11 meV, the origin of which is likely not related to a vibrational excitation—see Section 4.3 for discussion. From Figure 2b and Table S3 in the SI, we see that the HR and HT vibrational modes change between μ_4_-O_2_ and μ_2_-O_2_ configurations. However, we notice that the highest calculated frequencies of both μ_4_-O_2_ (36.9 meV) and μ_2_-O_2_ (38.3 meV) correspond to the out-of-plane HR, while the experimentally observed peak at 38.0 ± 0.1 meV is in-between the values obtained from the DFT-derived configurations. With this in mind, we correlate the vibrational frequencies of the same normal modes between μ_4_-O_2_ and μ_2_-O_2_ configurations in a Walsh diagram^48^—see Figure 5b. As the correlation parameter, we use the rotation angle of the molecular oxygen configurations found with respect to the Ag(100) lattice—μ_4_-O_2_ and μ_2_-O_2_ are defined at 0 and 90°, respectively. The experimentally observed μ_3_-O_2_ configuration has a small tilt angle of ∼20.7° with respect to μ_4_-O_2_ configuration at the 4-fold hollow site. We estimate this angle based on STM topographies, assuming that the O atoms are well aligned with the bright features of the image in Figures 1 and 3b,c. From the Walsh diagram in Figure 5b, we can unambiguously identify the ∼22 and ∼38 meV excitations to an in-plane HT and an out-of-plane HR, respectively. The in-plane HR mode, which we assigned independently based on action spectra, is also consistently correlated with this method. While the correlation matches for all similar excitations, the excitation at ∼11 meV cannot be consistently identified with any of the proposed vibrational normal modes.

Oxygen at Ag(100) was studied with high-resolution electron energy loss spectroscopy (HREELS) by Rocca, Gambardella, and co-workers.^24^ In their work, the electron beam cutoff covered any spectral features below ∼25 meV such that no peaks at 11 and 22 meV were observed. Two overlapping peaks were identified around 32 meV and both assigned to the out-of-plane HT of two different chemisorbed species. Due to the low spectral resolution of previous work (∼4 meV), we believe that the observed peaks correspond to those found in the IETS—Figure 2. We assign the vibrations in that range to the in-plane and out-of-plane HR and disagree with the assignment of the peaks around 32 meV from the previous work. Interestingly, a weak peak has been observed at 63 meV in HREELS, which is quite close to the threshold for the one-electron process (69.3 meV) seen in the rotation rate analysis. Our model potential simulation predicts a vibrational overtone (excitation from ground vibrational state to second excited state) of the in-plane HR around 60 meV. It is possible that the previously observed peak is related to such a vibrational overtone, the intensity of which would benefit from the strong anharmonicity of the rotational potential. Although previous work suggested the presence of μ_2_ and μ_4_ configurations, we think that the previously seen species are in fact μ_3_-O_2_ and μ_4_-O_2_ due to their close lying vibrational frequencies—see Figure 5b—explaining for example the doublet around 32 meV.

### 4.3 Discussion on the Nature of μ_3_-O_2_ Configuration

A very important aspect that distinguishes our work from—to the best of our knowledge—all previous studies on O_2_/Ag(100) is that we deposit molecules at ∼5 K surface temperature with an average normal translational energy of impinging oxygen molecules of ∼1 meV. At such low kinetic energies, O_2_ is expected to adsorb exclusively in a molecular state that has no barrier to adsorption. Such a state is often referred to as a “precursor state”, which is typically a poorly defined molecular state of oxygen, considered crucial for the thermal dissociation of oxygen molecules on many metals.^14,17^ This can be a weakly bound molecule, i.e., physisorption, or a molecular chemisorption state that forms chemical bonds with the metal through orbital overlap. In our case, the low temperature of the Ag(100) crystal is likely to suppress any subsequent process that would happen after the adsorption of the molecule into such a precursor state. However, even under our conditions, we encounter O atoms emerging from O_2_ dissociation on the surface—see SI Figure S2. Despite that, we believe that the μ_3_-O_2_ configuration observed in this work represents a precursor state of oxygen that plays an important role in thermal dissociation.

Previous STM work from Morgenstern and co-workers has claimed to observe both physisorption and chemisorption of O_2_ molecules after surface dosing at a temperature of 60 K.^49^ Although our conditions should be ideal to observe physisorbed molecules, we cannot confirm those findings but can exclude that μ_3_-O_2_ is a physisorbed state. The strongest argument against the physisorption state is that vibrational frequencies calculated from DFT for μ_4_-O_2_ and μ_2_-O_2_ agree quite well with μ_3_-O_2_. This suggests that the adsorption characteristics are shared between the three species. The DFT-derived states have binding energies on the order of ∼1 eV and exhibit substantial variations in the bond lengths of the molecules (compared to O_2_ in the gas phase), see Table S3 in the SI, which is not possible for physisorbed species. We also find the net negative Bader charge for μ_4_-O_2_ and μ_2_-O_2_ to be 0.6*e* and 0.9*e*, respectively, suggesting significant charge transfer from the metal, which is not typical for physisorbed molecules. Although PBE and RPBE exchange-correlation functionals are known to neglect dispersion interactions, which are crucial for physisorbed molecules, we do not think that this is a major cause of the mismatch. Dispersion correction is weakly specific toward coordination environments and would rather support higher coordination numbers.

Due to self-interaction errors, GGA-DFT leads to strong delocalization of charge. This results in weaknesses for the description of charge transfer and the charge distribution within the molecule, making the experiment-theory disagreement regarding the adsorption of O_2_ likely. To test whether an O_2_ anion can be energetically stable at Ag(100), we employ a simple model inspired by the work of Wodtke, Auerbach, and co-workers.^50^ To a first approximation, we may consider that the binding energy difference between the neutral and anionic state is only given by the Coulomb interaction of the charge and its image-charge for the adsorbed anion. In that case, the energetic stability of a negatively charged molecule at a metal surface Δ*E*, will be determined only by the work function of the metal Φ, the molecule’s electron affinity EA, and the Coulomb energy *E*_C_.

**Figure 6:**
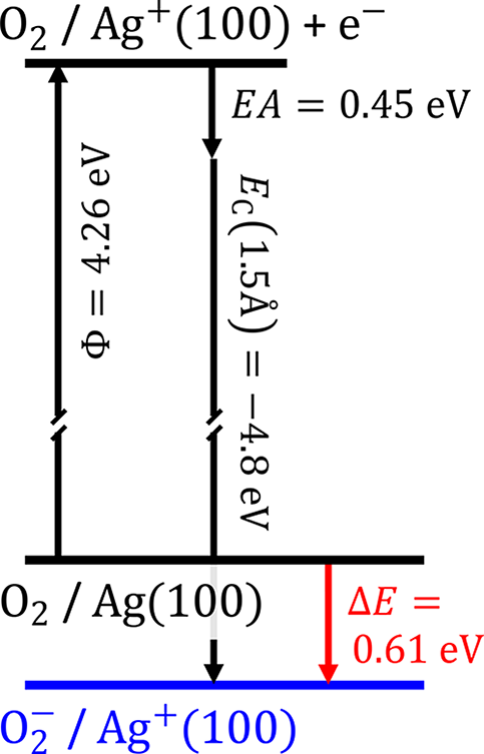
Simplified energy diagram^50^ demonstrating that an O_2_ anion can be more stable than a neutral molecule on Ag(100). The Coulomb energy is estimated at a distance of 1.5 Å, which is a typical bond distance for small molecules on surfaces.

The energy diagram for O_2_ adsorption on Ag(100) is sketched in Figure 6. Within this simple model, the anionic state would be 0.61 eV more stable than the neutral state, demonstrating that an O_2_ anion is energetically feasible. Although the energetics will be more complicated in reality, experience shows that systems with Φ – EA < 7 eV are prone to substantial charge transfer, which results in GGA-DFT^4^ becoming less reliable. The O_2_/Ag(100) system has Φ – EA = 4.21 eV, which is a particularly low difference, and will likely suffer from such issues as well.

The broad feature observed in IETS around 11 meV could be an indication of the presence of the anion as well. O_2_^–^ in the gas phase has a ^2^Π_Ω_ electronic ground state, for which spin degeneracy is lifted due to spin–orbit coupling. The energy difference between  and  states is 20 meV for the unperturbed anion.^31^ On the metal, the orbital angular momentum may be altered or quenched, as known for single transition metal atoms,^51,52^ due to orbital overlap with the metal. However, the orbital angular momentum is closely related to the chemical bond, and since it remains intact, the transition between different spin–orbit states may still be expected in a similar order of magnitude as in the gas phase. Such a transition would likely have a shorter lifetime than a vibrational transition, as it couples more closely to the electron bath, which may explain why the peak is ∼5 times broader than the vibrational transitions. At this stage, this interpretation is speculative and would require more careful experiments and explicitly correlated wave function theory to prove this point.

## 5 Conclusions and Outlook

We have identified and characterized an unexpected adsorption state of molecular oxygen—μ_3_-O_2_—on Ag(100). In this state, one of the O atoms of the molecule binds to the bridge site and the other O atom coordinates toward an opposite on-top site, inducing an asymmetric configuration where the molecule is connected to three Ag atoms simultaneously. GGA-level DFT calculations using the PBE and RPBE exchange-correlation functional were unable to identify μ_3_-O_2_ as a minimum. These calculations identify O_2_ at the 4-fold hollow and 2-fold bridge sites as minima only.

With IETS, we identified three vibrational excitations up to ±50 mV, which we assigned to out-of-plane HR, in-plane HR, and in-plane hindered translation—see Table 1. We also found that tunneling electrons promote a configurational change of the μ_3_-O_2_, with the threshold for this process coinciding with the excitation of the in-plane HR mode. This electron-induced configurational change of the molecule is closely related to a rotation on the surface, whereby the O atom coordinating to the bridge site jumps to a neighboring bridge site, while the O atom coordinating to the top site keeps—in good approximation—its position. We determine the barrier for this process to be 69.3 meV. Using a simple one-dimensional rotational potential model, we confirm the experimental finding of the presence of a two-electron and a one-electron process dominating the rotational isomerization—see Table 1.

**Table 1:**
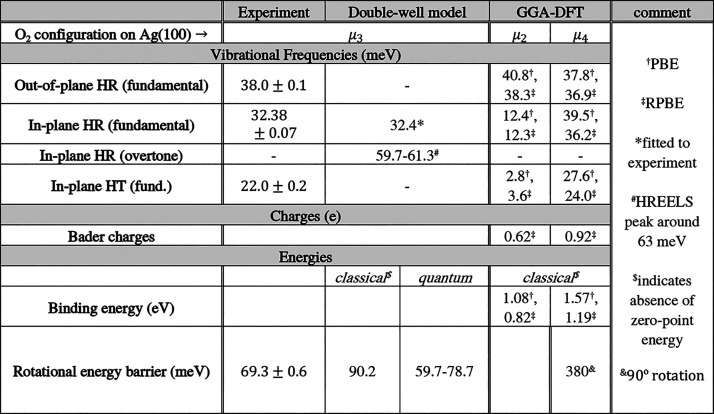
Summary of Vibrational Frequencies and Rotational Barriers Obtained in This Work from Experiment and Theory

We speculate that the observed μ_3_-O_2_ configuration might be a formal molecular oxygen anion that, based on simple arguments, might be energetically more stable than the neutral molecule. This would also explain the experiment-theory mismatch, which is likely to emerge as a consequence of poor description of charge transfer at GGA-level DFT.

The failure of GGA-DFT to identify the experimentally observed μ_3_-O_2_ adsorption might also have an impact on the dissociation of molecular oxygen on Ag(100). Recent molecular dynamics simulation for this reaction^21^ failed to reproduce the experimentally observed large O atom separation, which might conceivably be due to DFT-related inaccuracies in describing the relevant PES. Resolving the puzzle presented in this work could reveal some fundamental issues that cause the discrepancy between theory and experiment in the dissociation as well.

From the experimental point of view, μ_3_-O_2_ could still be better understood. For example, magnetic field-dependent IETS^53^ may identify the nature of the ±11 mV excitation, which we currently speculate emerges from the transition between spin–orbit coupled states of a surface-bound O_2_ anion. Also, Kelvin probe force microscopy^54^ could be used to identify the charge and the charge distribution within the molecule. Furthermore, the recently developed atomic-scale single-molecule sensors could be used to identify the spin-state of an oxygen molecule on Ag(100).^55−57^ Experimental methods that are able to reveal the electronic structure of molecules at surfaces are manyfold. This work may just be the beginning of an exciting avenue, where modern scanning probe methods will contribute to making surface chemistry a more exact science.

## Supporting Information Available

Additional STM topographies of single μ_3_-O_2_; large-scale topography of sample surface; interconversion between different μ_3_-O_2_ orientations on Ag(100); parameters for fits of inelastic tunneling spectra and rotation rates; additional telegraph noise data; additional discussion for rotational rate analysis; details for double-well rotational potential modeling with parameters; minimum energy pathway calculations for molecule rotation; calculated frequencies and geometries; and additional references (PDF)
