# Supplementary material for: Characterization of an Unexpected μ3 Adsorption of Molecular Oxygen on Ag(100) with Low-Temperature STM
Source: J Phys Chem C Nanomater Interfaces. 2024 Dec 31;129(2):1110–9. doi: 10.1021/acs.jpcc.4c06572 (PMC11744782; doi:10.1021/acs.jpcc.4c06572)
Supplement: Supplementary file 1 — jp4c06572_si_001.pdf [file jp4c06572_si_001.pdf]

Supporting Information for Publication

for

Characterization of an Unexpected μ_3 Adsorption of Molecular Oxygen on Ag(100) with Low-Temperature STM

Merve Ercelik[1,2], Andrés Pinar Solé[1,2], Liang Zhang[3], Piotr Kot[1,2], Jinkyung Kim[1,2], Jungseok Chae[1,2], Lukas E. Spree[1,2], Hua Guo[3], Andreas J. Heinrich[1,2] *, Yujeong Bae[1,2,†] **, Dmitriy Borodin[1,2] ***

[1]*Center for Quantum Nanoscience, Institute for Basic Science, Seoul 03760, South Korea*

[2]*Ewha Womans University, Seoul 03760, South Korea*

[3]*Department of Chemistry and Chemical Biology, Center for Computational Chemistry, University of New Mexico, Albuquerque, New Mexico 87131, USA*

[†]Present address: *EMPA, Swiss Federal Laboratories for Materials Science and Technology, Nanotech at Surfaces Laboratory, Dübendorf* 8600, *Switzerland*

Email: *: heinrich.andreas@qns.science, **: yujeong.bae@empa.ch, ***: borodin.dmitriy@qns.science

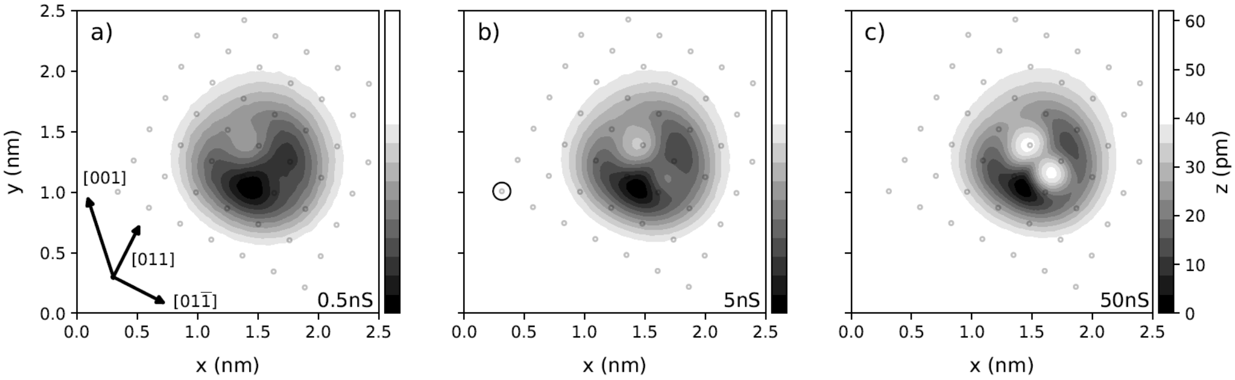

Figure S1. Constant current STM topography images of a single oxygen molecule on the Ag(100) surface under different tunneling current conditions. The images show one of the eight possible rotational conformers of the oxygen molecule on the surface with respect to the Ag lattice under different conductance conditions. The small circles represent the positions of Ag atoms. The large circle (in b)) is the positional uncertainty of the lattice with respect to the molecule. Each image was taken at a constant bias voltage of -10 mV while changing the tunneling current to (a) 5 pA, (b) 50 pA, (c) 500 pA.

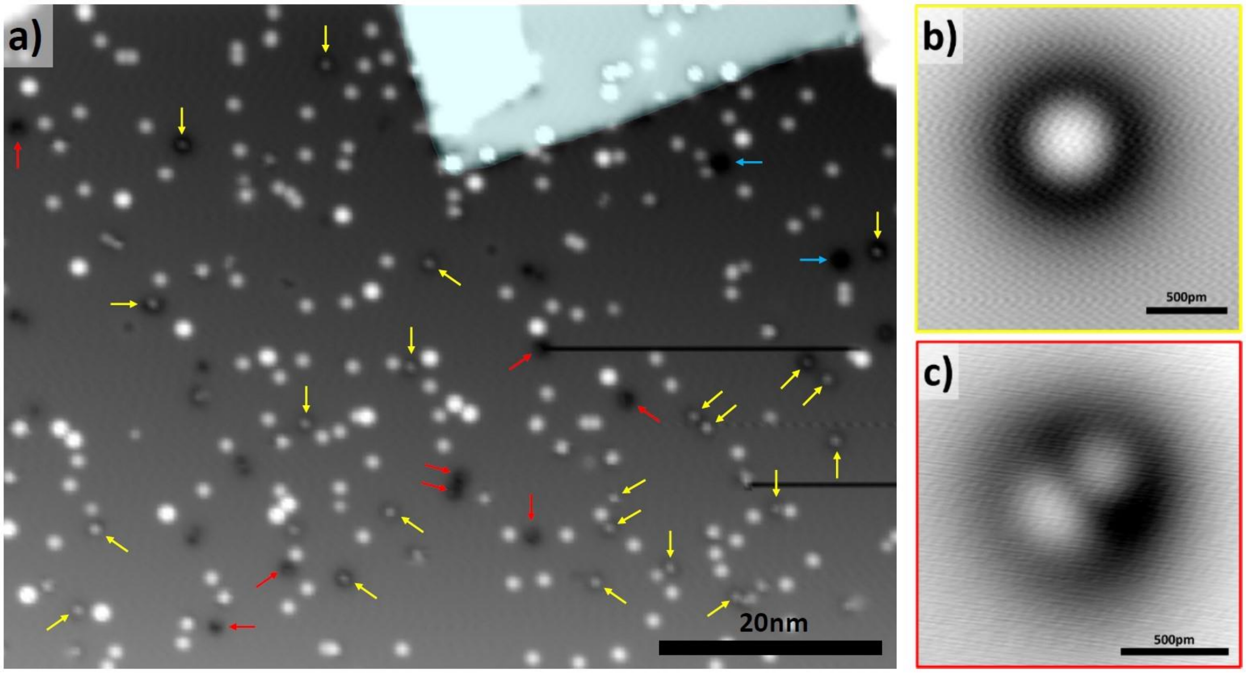

Figure S2. a) Large scale STM topography (-100 mV, 10 pA) of our Ag(100) sample after O_2 deposition. Prior to O_2 deposition the sample contained MgO thin films (highlighted in blue), Fe atoms (small atoms on silver), Ti atoms (large atoms on silver) and Nickelocene molecules (not visible on the image, due to very low concentration). Yellow arrows indicate O-atoms emerging after O_2 dissociation, red arrows indicate μ_3-O_2 molecules and blue arrow indicates an unknown species which was not present prior to O_2 deposition. The ratio of O-atoms to μ_3-O_2 is approximately 3:1. We speculate that the unknown species (blue arrow) may be either μ_2 or μ_4 configuration of O_2 that is predicted by GGA-DFT. However, due to their rare occurrence at our experimental conditions (10-20 times less than μ_3-O_2) we cannot make further conclusions on that. b) Single O-atom topography (-50mV, 10pA), c) Single μ_3-O_2 topography (-12mV, 250pA).

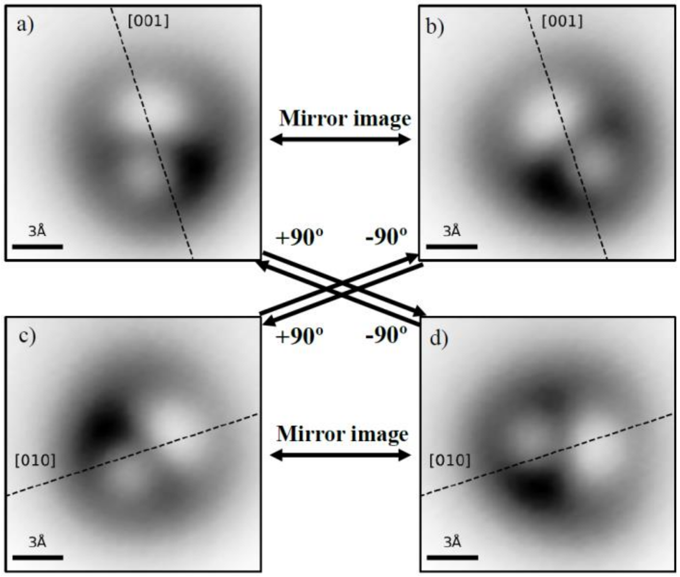

Figure S3. Four out of the eight possible rotational configurations of a single oxygen molecule on the Ag(100) surface are shown here. These configurations include their mirror images and 90º rotations. All images are taken at a bias voltage of -10 mV and 100 pA tunneling current. (a) and (b) are mirror images of each other, as are (c) and (d). (a) and (d), as well as (b) and (c), can be interconverted by 90º rotations into each other.

Table S1. The parameters of the model (Eq. 1) used to fit the dI/dV spectrum in Fig. 2.

	i = 1	i = 2	i = 3	i = 4
A_i (10^{-4} G_0)	0.178 ± 0.004	0.032 ± 0.003	0.086 ± 0.003	0.047 ± 0.003
B_i (10^{-4} G_0)	0.156 ± 0.004	0.023 ± 0.003	0.085 ± 0.003	0.037 ± 0.003
$\varepsilon_{0,i}$ (meV)	11.06 ± 0.12	21.96 ± 0.20	32.38 ± 0.07	37.97 ± 0.12
$T_{eff,i}$ (K)	28.83 ± 1.56	5.49 ± 2.10	5.325 ± 0.650	4.16 ± 1.12
c (10^{-4} G_0)	2.039 ± 0.002			

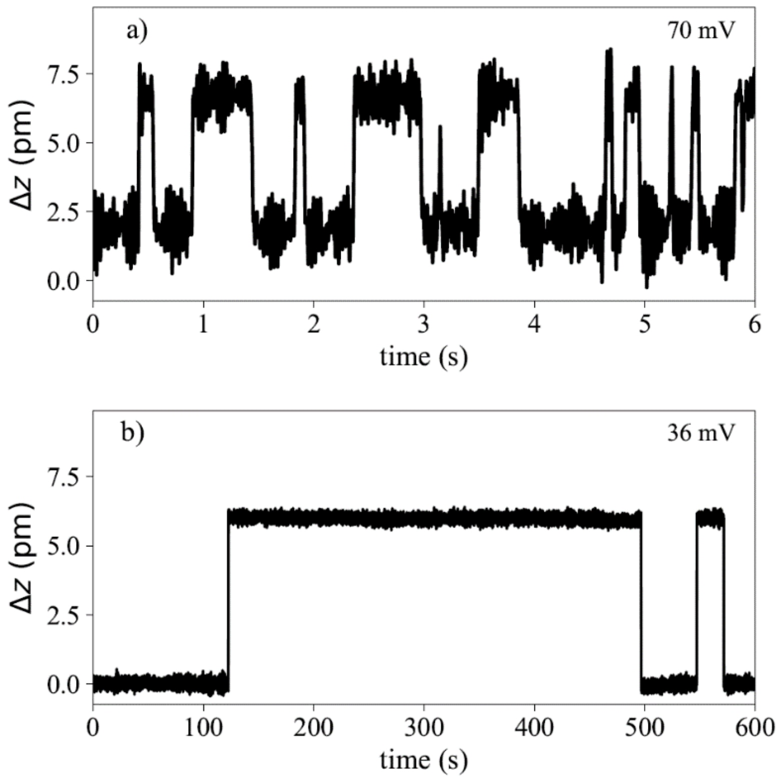

Figure S4. Telegraph noise of a single μ_3-O_2 on Ag(100) measured at a constant tunneling current of 1 nA for bias voltages of 36 mV and 70 mV. The position of the STM tip on the molecule is as shown in Fig. 3 (b) and (c). The time-dependent data show the relative z height difference and switching between two rotational states. Low switching rate is observed at low bias voltages, while increasing the bias voltage results in a higher switching rate. Notice the 100-fold difference between the timescales in panel a) and b).

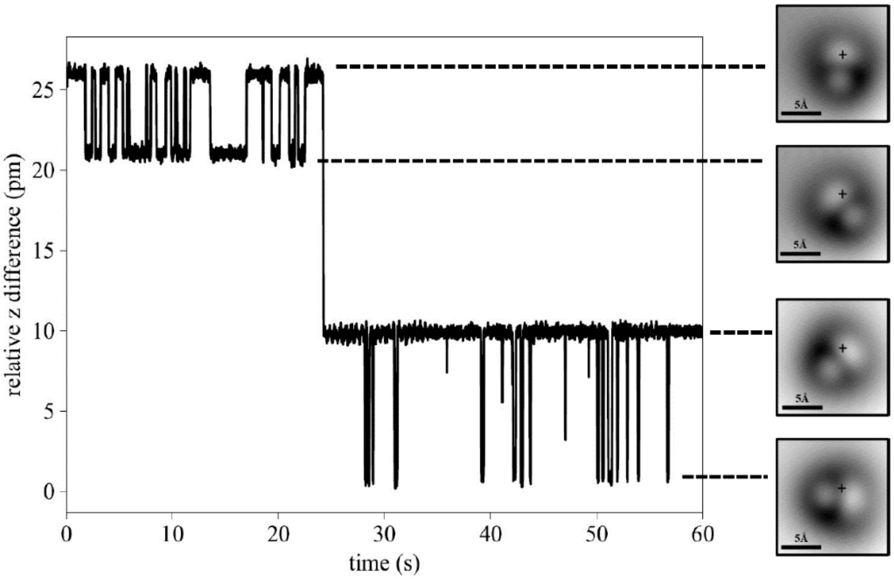

Figure S5. Telegraph noise of a single μ_3-O_2 molecule on Ag(100) measured at 60 mV and 1 nA. The time-dependent data illustrate the relative z height difference and switching between four rotational states. Constant current topography images, taken at -10 mV and 100 pA, corresponding to each rotational state are shown. The plus sign in the images represents the STM tip position during the measurement.

Table S2. The parameters of Eq. 2 for the rotation rate in one-electron and two-electron processes

	One-electron process (i = 1)	Two-electron process (i = 2)
n	1	2
$V_{0,i}$ (mV)	69.3	32.38
a_i ($\mathrm{mV^{-1}nA^{-1}s^{-1}}$)	2	$1.323 \times 10^{-3}\ \mathrm{mV^{-1}\ nA^{-1}}$

Sec. S1: Consideration of alternative explanations for the action spectra

We exclude the possibility of a one-electron or a three-electron process below 69.3 mV is dominating the observed rates—see Fig. S6. We also considered introducing an additional branch in the rotational rate around 38 mV, which would coincide with the vibrational transition observed in dI/dV. However, even using the degree of the electron process as an empiric parameter, we were unable to get a satisfying agreement with the voltage and the current dependence of the rotation rate—see Fig. S7. We explain the deviations observed around 38 mV by electrons channeling into a vibrational excitation rather than a rotational process. Close to the resonance, we observe a dip that is absent before and after the 38 mV transition, supporting our hypothesis. We conclude that the analysis presented in the main text is the most likely one.

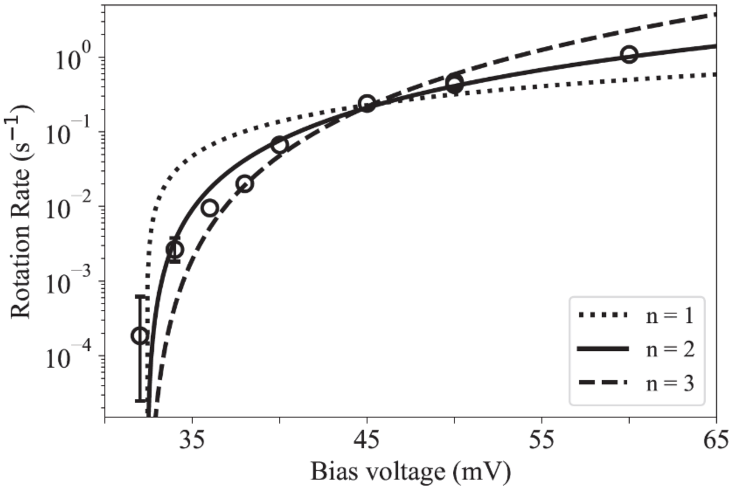

Figure S6. The rotation rate as a function of bias voltage is fitted according to one-electron, two-electron, and three-electron processes. Except for the two-electron process, the bias voltage dependence cannot be fitted satisfyingly.

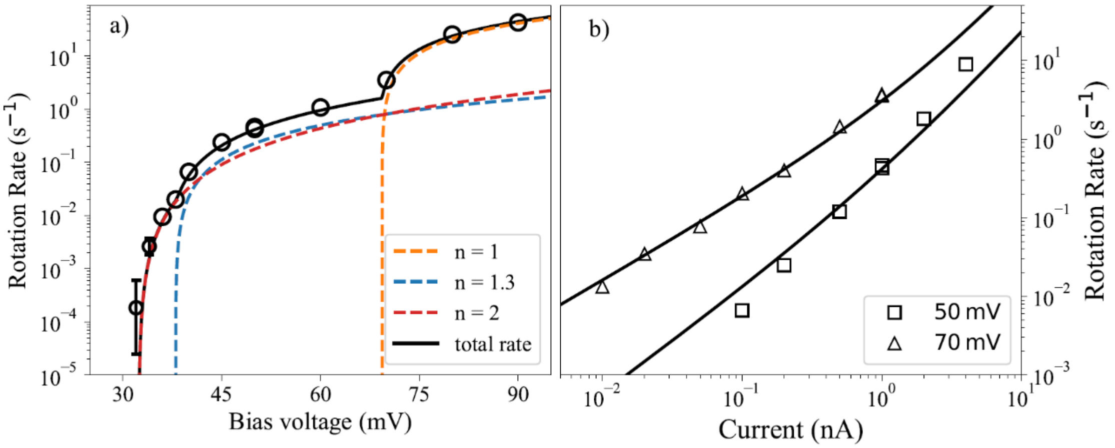

Figure S7. Rotation rates of oxygen molecule on Ag(100) as a function of bias voltage and tunneling current. When the fit is performed based on data at 38 mV, we observed three branches in bias voltage plot, while the rotation rate vs current graph cannot be accurately obtained.

Sec. S2: Set-up of 1D rotational potential and eigenenergy determination

From Fig. 3 (b) and (c), we learn that the model potential should have a mirror symmetry with respect to the rotation coordinate. Analyzing the difference in the rotation angle between the two configurations—see Fig. S8—allows us also to parametrize the angle between the two minima. We use the lowest order polynomial needed to produce a double-well potential. The parameters of the polynomial function are redefined to express the potential in terms of the rotation angle between minima Δ and the classical barrier for rotation W in the following way:

$$V_{\mathrm{rot}}(\varphi) = \frac{16\,W}{\Delta^4}\varphi^4 - \frac{8\,W}{\Delta^2}\varphi^2 + W \tag{S1}$$

We define the angle φ as the angle between the molecule bond and the axis of the mirror image plane ([001] or [010]). We obtain the eigenenergies for this potential by solving the nuclear Schrödinger equation:

$$\left(-\frac{\hbar^2}{2I}\frac{\mathrm{d}^2}{\mathrm{d}\varphi^2} + V_{\mathrm{rot}}(\varphi)\right)\psi = E\psi \tag{S2}$$

with the discrete variable representation—see Ref. 1. Due to the interaction of the molecule with the surface, we consider that the rotation will not be fixed around the molecule's center of mass but might be shifted along the internuclear distance of the molecule. Therefore, we define the moment of inertia as:

$$I = m_{\mathrm{O}}(\alpha\, r_0)^2 + m_{\mathrm{O}}((1-\alpha)\, r_0)^2 \tag{S3}$$

with m_{O} as the mass of the O-atom and r_0 as the internuclear distance of the O_2 molecule, which we assume is 1.2 Å. The parameter α defines a position at the internuclear axis which acts as an effective center of mass. If $\alpha = 0$, the molecule rotates with one of the O-atoms being held in place; for $\alpha = 0.5$, the molecule rotates around its center. We estimate the alpha parameter to be around 0.3—see Fig. S8.

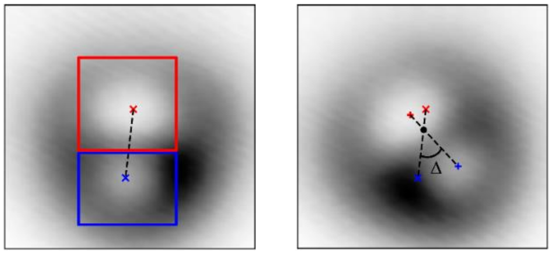

Figure S8. Determination of the rotation angle and intersection point of the rotation required to model the nuclear eigenstates. (a) The red and blue boxes are used to identify the maximum points of the bright lobes. (b) The angle between two configurations is denoted as Δ, while the dot indicates the intersection point of the rotation. We find the intersection point of the rotation to be at $\alpha = 0.3$ times the bond length—see Eq. S3.

The classical barrier for isomerization W is optimized such that the first vibrational transition matches with 32.4 meV found in the experiment—the corresponding results for the potential are shown in Fig. 5 (a) and its parameters are summarized in Table S4.

The wavefunctions of the eigenstates below the classical barrier, are transformed to localized configurations '$+$' and '$-$' through linear combination of eigenstates with same energy. The basis for the localized states are wavefunctions that are delocalized across two mirror image conformers, which directly result from diagonalization of the Hamiltonian.

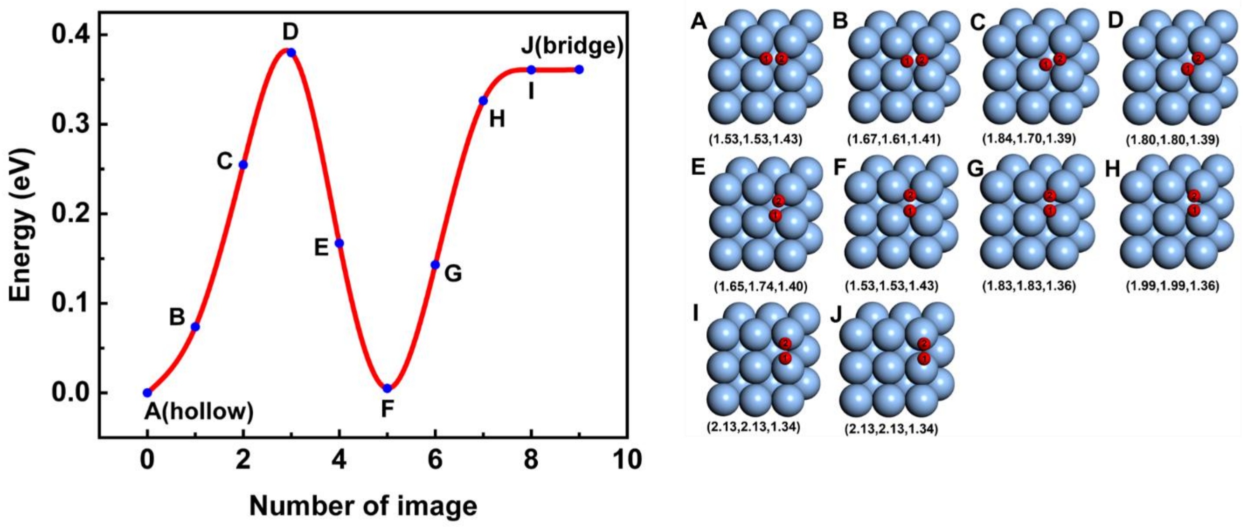

Figure S9. Isomerization minimum energy path from the 4-fold hollow site oxygen molecule (μ_4-O_2) to a bridge site molecule (μ_2-O_2), determined by Climbed-Image Nudged Elastic Band (CI-NEB) method. For A-J, the first two entries in parenthesis indicate the z-height of the O-atom and the third entry is the bond length of the oxygen molecule in Å. Images C and E are similar to the μ_3-O_2 structure found from the experiment, but are not stable local minima. The barrier for a 90° rotation of μ_4-O_2 is 0.38 eV. The barrier for μ_2-O_2 to μ_4-O_2 isomerization is only 19 meV. We find that adsorption to both configurations is barrierless.

Table S3. Vibrational frequencies (in meV) of the adsorbate at the four-fold hollow and two-fold bridge sites, the calculated bond length (*r*), the distances between the oxygen atoms and the first layer of the surface (Z_1, Z_2), the adsorption energies (*E*, not corrected for zero-point energy) of the adsorbate, and Bader charge values transferred from the Ag surface to oxygen at the hollow and bridge sites, calculated using the PBE and RPBE functionals.

	μ_2		μ_4	
	**PBE**	**RPBE**	**PBE**	**RPBE**
**Vibrational frequencies (meV)**				
**O-O stretch**	129.5	128.4	98.4	97.6
**Out-of-plane hindered rotation**	40.8	38.3	37.8	36.9
**In-plane hindered rotation**	12.4	12.3	39.5	36.2
**Hindered translation along x**	2.8	3.6	18.5	17.5
**Hindered translation along y**	17.5	15.9	27.6	24.0
**Hindered translation along z**	30.3	26.6	28.7	26.3
**Other calculated results**				
r **(Å)**	1.34	1.34	1.43	1.43
Z_1 **(Å)**	2.07	2.13	1.46	1.53
Z_2 **(Å)**	2.07	2.13	1.46	1.53
E **(eV)**	1.08	0.82	1.57	1.19
**Bader charge (*e*)**	0.61	0.62	0.93	0.92

Table S4. Parameters of 1D rotational potential model used to generate Fig. 5 (a).

**Parameters**	**Value**
Potential barrier W	90.2 meV
Bond length r_0	1.2 Å
Intersection point of rotation α	0.3
Angle difference between conformers Δ	48.6°

Additional references

1. Borodin, D., et al., Quantum Effects in Thermal Reaction Rates at Metal Surfaces. *Science* **2022**, *377*, 394-398.
